# Comparing the effect of two different intraocular lenses on optical aberrations in bilaterally operated eyes for cataract

**DOI:** 10.12669/pjms.294.3607

**Published:** 2013

**Authors:** Esra Ayhan Tuzcu, Kuddusi Erkilic, Betul Bulut, Nilufer Ilhan

**Affiliations:** 1 Esra Ayhan Tuzcu, Assistant Professor, Department of Ophthalmology, Medical Faculty of the Mustafa Kemal University, Hatay, Turkey.; 2 Prof. Kuddusi Erkilic, Professor, Department of Ophthalmology, Medical Faculty of the Erciyes University, Kayseri, Turkey. Department of Ophthalmology, Medical Faculty of the Mustafa Kemal University, Hatay, Turkey.; 3 Dr. Betul Bulut, Department of Ophthalmology, Elmadag State Hospital Ankara, Turkey. Department of Ophthalmology, Medical Faculty of the Mustafa Kemal University, Hatay, Turkey.; 4 Nilufer Ilhan, Assistant Professor, Department of Ophthalmology, Medical Faculty of the Mustafa Kemal University, Hatay, Turkey.

**Keywords:** Cataract, Intraocular lens, Optical aberration

## Abstract

***Objective:*** To assess high order and spherical aberrations results of hydrophobic acrylic AMO Sensar AR40E and hydrophobic acrylic Alcon AcrySof SA60AT intraocular lenses after implantation in cases with bilateral cataract.

***Methods:*** Cases diagnosed as bilateral cataract were included in the study and preoperative aberration measurements were recorded by using Nidek OPD SCAN-ARK 1000. Groups were created by implanting AMO Sensar AR40E to one eye of the patients, while Alcon AcrySof SA60AT into the other in a prospective and randomized manner. Aberration measurements were recorded after one and two months of surgery.

***Results:*** Overall, 40 eyes in 20 patients (11 women and 9 men) were included in the study. All patients underwent bilateral phacoemulsification surgery due to cataract. There were 20 eyes in both groups. Mean age was 62.4 (range: 31-82) years. There was no significant difference in aberrations recorded before surgery and one and two months after surgery in both groups. (p<0.05).

***Conclusion:*** There was no difference among spherical intraocular lenses used in this study.

## INTRODUCTION

Cataract is one of the main preventable causes of blindness.^1^^-^^3^ Surgery is still the only cure option in cataract management.^4^ Pathogenesis of cataract is unclear. Over time, thickness and weight of lens increases, while the ability of accommodation decreases.^5^^,^^6^ In cases with cataract, changes in refraction causes high-order aberrations. Refractive index (RI) increases due to nuclear cataract. As a result, spherical aberration increases.^7^ Quality of lens implanted into the eyes is the most important factor which will determine final visual quality. 

Spherical intraocular lenses (IOLs) cause spherical aberration in pseudophakic eyes, where it has little effect on other aberrations. Aberrations vary depending on the dioptric power, optical diameter, material, design and RI of the IOLs. In the phakic eyes, cornea and lens are the two optical elements determining quality of retinal image. In young individuals, positive spherical aberration caused by cornea is neutralized by negative spherical aberration in the lens. However, in elderly individuals, shift towards positive in spherical aberration related to lens increases total aberration of the eye.^8^^,^^9^

The aim of this study was to assess the optical aberration results of hydrophobic acrylic AMO Sensar AR40E and hydrophobic acrylic Alcon AcrySof SA60AT intraocular lenses after implantation in cases with bilateral cataract.

## METHODS

This study was conducted in Ophthalmology Department of Erciyes University, Medicine School in a prospective and randomize manner. All patients gave informed consent. The study was carried out in accordance to Helsinki Declaration Criteria.

***Patient Selection: ***A complete history was obtained before surgery. All patients underwent vision examination, biomicroscopic examination, measurement of intraocular pressure, fundus examination and B-scan ultrasound. Patients previously underwent intraocular surgery, those with corneal opacity or irregularity, those with preoperative and postoperative complications, and those lost in follow-up were excluded. Overall, 40 eyes in 20 patients (11 women and 9 men) were included to the study. There were 20 eyes in both groups. Mean age was 62.4 (range: 31-82) years.

***Intraocular lenses: ***We used spherical Sensar AR40E (group 1) and spherical AcrySof SA60AT (group 2) lenses. Both lenses have hydrophobic acrylic structure with an optical length of 13 mm and optical diameter of 6 mm. Sensar AR40E is a three-pieces lens with an angle of 5° and RI of 1.47, while AcrySof SA60 AT is a mono-block lens without angle and it has a RI of 1.55. In all cases, intraocular lenses were implanted using similar techniques after phacoemulsification surgery by a single surgeon.

***Preoperative evaluation and postoperative follow-up: ***A two-month follow-up was scheduled to monitor aberration. Aberrations were measured at 4 mm optical zone before surgery and on the month 1 and 2 after surgery by using Nidek OPD SCAN-ARK 1000. On the month 1 and 2, eyes were dilated after measurements of aberration and position of IOLs in the capsule were evaluated.

***Statistical Analysis: ***All statistical analysis were performed by using SPSS for Windows 10.0. Paired t test and Wilcoxon rank test were used for comparisons. P<0.05 was considered as significant.

## RESULTS

**Graph.1:**
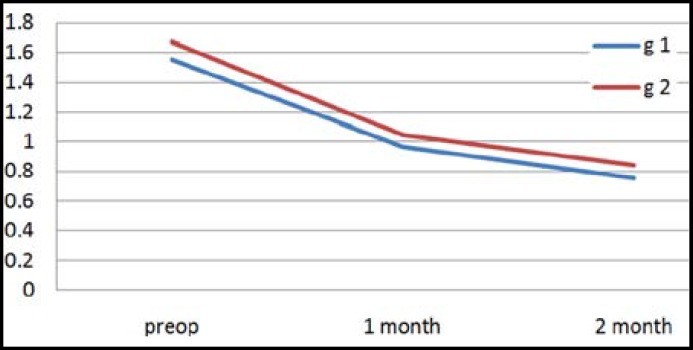
Average values for HO aberration in groups according to periods

**Graph.2:**
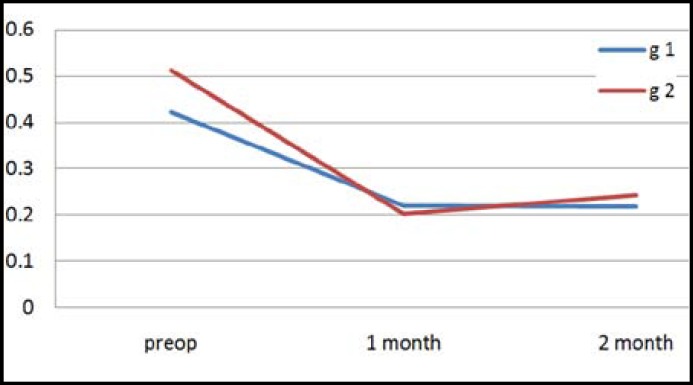
Average values of spherical aberration in groups according to periods

In the present study, inter-group or intra-group comparisons were performed for high-order (HO) and spherical aberration values recorded before surgery and on the month 1 and 2 after surgery. There was no significant difference between group 1 and 2 in terms of HO aberration values recorded before surgery and on the month 1 and 2 after surgery (p>0.05) (Table-I) (Graph.1). There was no significant difference between group 1 and 2 in terms of spherical aberration values recorded before surgery and on the month 1 and 2 after surgery (p>0.05) (Table-I) (Graph.2). No preoperative or postoperative complication occurred in any case. No decentration was detected in any IOL implanted.

**Table-I:** Preoperative and postoperative aberration values of intraocular lenses

	*Sensar AR40e *	*Acrysof SA60AT *	p*
*HO aberration*			
Preop	1.549	1.672	0.795
1 month	0.969	1.042	0.799
2 month	0.755	0.839	0.427
*Spherical aberration*			
Preop	0.422	0.512	0.700
1 month	0.220	0.202	0.823
2 month	0.218	0.243	0.518

## DISCUSSION

The cornea and lens are the two optical elements determining quality of retinal image in the phakic eyes.^10^ In young individuals, positive spherical aberration caused by cornea is neutralized by negative spherical aberration in the lens.^10^ However, in elderly individuals, shift towards positive in the spherical aberration related to the lens increases total aberration of the eye. Since majority of cases with cataract are elderly patients, association of cataract and age further increase the aberration; thus, resulting in reduced quality of retinal image. In the cataractous lens, local refractive changes cause increase in HO aberration. In our study, we detected that there was a significant decrease in HO aberration values recorded on the month 1 and 2 in group 1. In agreement with our study, there are studies reporting increased HO aberration values in cases with cataract in the literature.^7^^,^^11^

It was found that HO aberration values recorded on the months 1 and 2 after surgery was lower in Sensar AR40E group compared to AcrySof SA60AT group, although the difference didn’t reach statistical significance. Villarodona et al^12^ found that HO aberrations was higher in IOLs with higher RI in pseuodphakic eyes. Martin and Sanders^13^ compared HO aberration values of the eyes in which STAAR Collamer (RI=1.44), STAAR silicone (RI=1.41), Sensar AR40E (RI=1.47) and AcrySof SA60AT (RI=1.55) lenses were implanted. Authors found that AcrySof SA60AT lens caused more aberration than STAAR Collamer lens, but the difference wasn’t statistically significant.^13^ The RI of AcrySof SA60AT lens is higher compared to Sensar AR40E lens used in our study. In the light of data in literature, the difference (statistically insignificant) in HO aberration values between these two lenses may be related to difference in RI. 

In our study, no significant difference was found between Sensar 40E and AcrysSof SA60AT in terms of spherical aberration values recorded before surgery and at month 1 and 2 after surgery. However, it was found that spherical aberration values recorded at month 1 after surgery was lower in AcrySof SA60AT lens than those in Sensar AR40E, while it was lower in Sensar AR40E than AcrySof SA60AT at month 2 after surgery. However, the differences were insignificant (p>0.05). These differences may be related to lower RI and forward angulation of haptics in Sensar 40E lens. As the RI increases, anterior curvature of the lens decreases causing increased spherical aberration. In a study by Rohart et al^14^, in which three-pieces hydrophobic acrylic AcrySof Ma60AC lenses (RI=1.55) and mono-block hydrophobic acrylic XLSTABI lenses (RI=1.46) were implanted to eyes with cataract, it was found that AcrySof MA60AC lens caused more spherical aberration. Authors attributed this finding to higher RI of AcrySof MA60AC lens than the other lens. Taketani et al^15^ found that forward-angled AcrySof lenses cause less aberration in the optical zone of 6 mm, whereas Erie et al^16^ found that forward-angled lenses prevent surface glare. These data in literature may explain the lower spherical aberration values recorded with Sensar AR40E, which has a forward haptic angulation, than the other lens. 

Spherical aberrations of intraocular lenses increase, as its optical power increases. Bellucci et al^17^ measured spherical aberrations of AcrySof MA60BM lenses with different dioptres and found that spherical aberration increased as the amount of dioptre increases. Bellucci et al^17^ observed that aberration values were excessively increased after implantation of +30 Dpt lens in a patient. Padmanabhan et al^18^ found that higher dioptres of AcrySof MA60BM lenses induced spherical aberration. In our study, there was no difference between eyes in terms of dioptric power of lenses.

In recent years, spherical lenses used in our study have been compared to aspherical lenses in several studies. No difference was detected in HO aberration levels between spherical and aspherical lenses. However, spherical aberration values were found to be significantly lower in aspherical lenses than spherical ones.^19^^-^^24^ Because the aspherical lenses cause negative spherical aberration and compensate spherical aberration of cornea as in young individuals. However, spherical lenses cause more spherical aberrations than aspherical lenses, as they don’t compensate positive spherical aberration in cornea in addition to its inherent aberration.

Our results showed that both Sensar AR40E and AcrySof SA60AT lenses significantly correct aberrations caused by cataract in eyes where they are implanted and do not cause a marked increase in spherical aberration. Although there was a difference in terms of correcting aberrations between these two lenses, this difference wasn’t found to be statistically significant. Although, of these lenses with spherical optical surface design, one is mono-block and other is three-pieces lenses, similar results were obtained as there isn’t excessive difference in terms of either margin characteristics or refractive index. Larger series are needed, demonstrating how spherical lenses affect HO and spherical aberrations.

## Authors Contributions:

KE, EAT conceived, designed and did statistical analysis & editing of manuscript.

EAT, BT & NI did data collection.

KE, EAT did review and final approval of manuscript.

## Notes

***Financial support and sponsorship:*** None.
